# The Innervation of the Salivary Glands

**Published:** 1882-01

**Authors:** 


### THE INNERVATION OF THE SALIVARY GLANDS.

The remarkably exact knowledge which we possess of the innervation of the submaxillary gland has been considerably extended by Aschenbrandt, of Cassel, who has investigated the nervous relations of the other glands of the same class; the character of the different secretions furnished by the parotid, sublingual, and submaxillary glands, respectively; and the reflex relations of these structures to certain other parts of the body, notably the conjunctiva. The experiments were made on carnivorous animals. The principal results are summarized in the *Lancet and Clinic* as follows:—Fluids that irritate the conjunctiva, cause salivation. The secretion of the parotid is influenced by the glosso-pharyngeal nerve, whilst the facial trunk has no share in salivation. The sympathetic is not influenced by irritation of the conjunctiva. All the three salivary glands participate in reflex salivation. The impression on the conjunctiva is reflected through the nuclei of the nerves. The character of saliva furnished by the different glands, and obtained by the various methods of reflex irritation, respectively, may be found described in the original paper in Pfluger's *Archiv*, Band xxv. s. 101.

